# A robust and versatile deep learning model for prediction of the arterial input function in dynamic small animal [^18^F] FDG PET imaging

**DOI:** 10.1186/s13550-026-01398-9

**Published:** 2026-03-09

**Authors:** Christian Salomonsen, Luigi T. Luppino, Fredrik Aspheim, Kristoffer Wickstrøm, Elisabeth Wetzer, Michael Kampffmeyer, Rodrigo Berzaghi, Rune Sundset, Robert Jenssen, Samuel Kuttner

**Affiliations:** 1 Department of Physics and Technology, UiT The Arctic University of Norway, Tromsø, Norway; 2 PET Imaging Center, University Hospital of North Norway, Tromsø, Norway; 3 Department of Clinical Medicine, UiT The Arctic University of Norway, Tromsø, Norway

**Keywords:** Dynamic positron emission tomography (PET), Small-animal PET, Arterial input function prediction, Deep learning, Prediction model

## Abstract

**Background:**

Dynamic positron emission tomography (PET) and kinetic modeling are pivotal in advancing tracer development research in small animal studies. Accurate kinetic modeling requires precise input function estimation, traditionally achieved through arterial blood sampling. However, arterial cannulation in small animals, such as mice, involves intricate, time-consuming, and terminal procedures, precluding longitudinal studies. This work proposes a non-invasive, fully convolutional deep learning-based approach (FC-DLIF) to predict input functions directly from PET imaging data, which may eliminate the need for arterial blood sampling in the context of dynamic small-animal PET imaging. The proposed FC-DLIF model consists of a spatial feature extractor that acts on the volumetric time frames of the dynamic PET imaging sequence, extracting spatial features. These are subsequently further processed in a temporal feature extractor that predicts the arterial input function. The proposed approach is trained and evaluated using images and arterial blood curves from [^18^F]FDG data using cross validation. Further, the model applicability is evaluated on imaging data and arterial blood curves collected using two additional radiotracers ([^18^F]FDOPA, and [^68^Ga]PSMA). The model was further evaluated on data truncated and shifted in time, to simulate shorter, and shifted, PET scans.

**Results:**

The proposed FC-DLIF model reliably predicts the arterial input function with respect to mean squared error and correlation. Furthermore, the FC-DLIF model is able to predict the arterial input function even from truncated and shifted samples. The model fails to predict the AIF from samples collected using different radiotracers, as these are not represented in the training data.

**Conclusion:**

Our deep learning-based input function offers a non-invasive and reliable alternative to arterial blood sampling, proving robust and flexible to temporal shifts and different scan durations.

**Supplementary information:**

The online version contains supplementary material available at 10.1186/s13550-026-01398-9.

## Introduction

Dynamic positron emission tomography (PET) plays a critical role in small-animal imaging by enabling *in vivo* visualization of tracer uptake over time. These measurements allow subsequent quantification of tracer transport or binding through kinetic modeling [1]. This is actively used as a tool for downstream tasks, for example in the development of new tracers, drugs, diagnostic procedures, and disease therapies [2–6]. However, a prerequisite for kinetic modeling is knowledge of the arterial tracer concentration over time, commonly referred to as the arterial input function (AIF) [7, 8].

Arterial blood sampling is considered the gold standard for AIF acquisition, but in preclinical settings it presents serious limitations. The procedure is technically complex, time-consuming, and terminal in mice due to the invasiveness of carotid cannulation. Furthermore, only a limited amount of blood volume can be withdrawn without altering animal physiology [9, 10]. These limitations make high-throughput and longitudinal studies infeasible and may further raise ethical concerns regarding excessive animal usage [11].

This has driven significant interest in non-invasive AIF estimation strategies. Population based input functions (PBIFs) averages time-activity curves (TACs) from demographically similar subjects [12]. They fail to capture individual variability and still requires at least one blood sample to scale the population curve. Image-derived input functions (IDIFs) extract TACs from vascular regions such as the left ventricle. However, they are prone to inaccuracies stemming from partial-volume effects, motion artifacts, and poor signal-to-noise ratios [9, 13–16]. Simultaneous estimation (SIME) approaches reduce the reliance on blood sampling by fitting both tissue kinetics and the AIF from multiple regions concurrently [17]. These require a predefined functional form for the AIF and still depend on at least one late-time blood sample to anchor the estimated curve to absolute tracer concentrations [18–21].

To address these limitations, several data-driven alternatives have been proposed. In our previous research, we developed machine learning-based methods for AIF estimation using extracted TACs of visible blood pools as input [22, 23]. While these methods eliminated the need for blood sampling, they relied on manually delineated regions of interest (ROIs) and post-imaging TAC extraction. This limited their scalability and integration into automated pipelines.

In more recent studies, it has been demonstrated that deep learning-based methods outperform more traditional machine learning-based methods, bypassing the need for handcrafted features and manual ROI delineation by prediction of the AIF directly from the dynamic PET image volume [24–27]. However, most of these methods adopt hybrid architectures that combine 3D convolutional layers to extract spatial information, and fully connected or recurrent layers, to process temporal dependencies. These architectural choices introduce key limitations. For example, fully connected layers necessitate fixed-length inputs, forcing all dynamic scans to be padded, truncated, or interpolated to a uniform number of time frames prior to inference [24, 25]. This increases computational overhead and may introduce interpolation artifacts, especially when acquisition protocols differ in temporal resolution or duration.

Recurrent-style models, such as LSTM-based networks [24, 27], attempt to model temporal dependencies explicitly. However, they often learn features tied to specific time indices, reducing robustness to timing shifts or variation in tracer arrival across subjects. Furthermore, several methods include *post hoc* fitting or model parameter regression [26, 27], adding assumptions and complexity to the pipeline. These constraints can hinder generalization and reduce flexibility in practical settings.

In this study, we propose a novel approach: Fully convolutional deep learning-based input function prediction (FC-DLIF), that overcomes these limitations by the exclusive use of convolutional operations over both spatial and temporal dimensions. The fully convolutional design allows the FC-DLIF to process input sequences of arbitrary length, eliminating the need for dense or recurrent layers, and removing dependence on fixed temporal input shapes. The FC-DLIF takes reconstructed 4D dynamic PET data (t, x, y, z) and directly predicts the AIF as a 1D time-activity curve. It does so without the need for manual ROI segmentation, TAC extraction, temporal resampling, or post hoc fitting. The FC-DLIF treats time as a learnable axis and detects temporal patterns such as peak onset, plateau, and washout tail, regardless of absolute frame positions. This makes the FC-DLIF inherently robust to timing variability and flexible across different imaging protocols. The architecture provides a streamlined, end-to-end pipeline for non-invasive AIF estimation suitable for diverse preclinical study designs.

We evaluate the FC-DLIF on a dynamic PET dataset of mice with paired arterial blood data, and show that our method generalizes across protocols with time-shifted inputs and different scan durations. By accurate, non-terminal, and non-invasive AIF estimation, the proposed method has the potential to reduce animal use, facilitating longitudinal studies, and simplify the workflow for kinetic-modeling, aligning with the 3Rs principles of animal research [11].

## Materials and methods

**Fig. 1:**
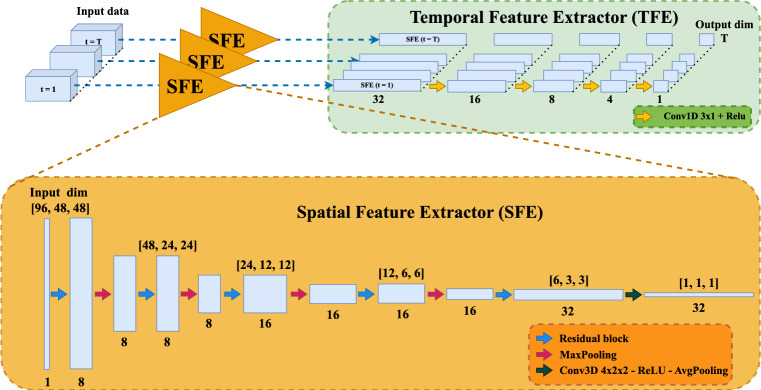
The proposed architecture consists of two parts: a 3D ResNet [28] acts as a spatial feature extractor (SFE), and a 1D convolutional network that acts as a temporal feature extractor (TFE). The first takes a single PET time frame, and reduces its dimensions to a vector of 32 features. All SFE-extracted vectors are stacked along the time dimension, which the second part of the model applies a series of convolutions on, until the vector dimension is reduced to 1. The final output of the model is a time series with the same length as the input data: The arterial input function

This section describes the data, the design of the proposed deep learning model, and its training procedure. Further details on data collection are provided in the Supplementary (Sec. S1).

### Dataset

The training dataset consisted of 70 dynamic $\mathrm {[^{18}F]FDG}$ PET scans with simultaneously measured AIFs of mice in ages 9 to 24 weeks, collected at UiT The Arctic University of Norway (UiT). The dataset included three different mouse strains: BALB/cJRj (N = 55), C57BL/6JRj (N = 8) and Balb/cAnNCrl (N = 7). Another 10 samples, collected with different tracers $\mathrm {[^{18}F]FDOPA}$ (N = 6) and $\mathrm {[^{68}Ga]PSMA}$ (N = 4), which were not included during model training, but used to evaluate the model performance on new, unseen tracers. Following PET imaging, dynamic image volumes were reconstructed into 42 time frames (1$\times$ 30 s, 24$\times$ 5 s, 9$\times$ 20 s, and, 8$\times$ 300 s) of whole-body data with shape 42$\times$96$\times$48$\times$48, and normalized into standardized uptake value (SUV) [29]. Detailed information on data acquisition and processing can be found in [30].

### Model architecture

Fig. 1 shows the architecture of the proposed model. It consisted of two parts: a spatial, and a temporal feature extractor. First, the data volume associated with each time step of the dynamic scan was passed through a 3D ResNet [28], called a spatial feature extractor (SFE), which extracted relevant spatial features while only reducing the spatial dimensions. From the input, each data volume went through a series of residual blocks interleaved with max-pooling layers. Then, a convolutional layer with a 4$\times$2$\times$2 cuboid kernel, was followed by an adaptive average-pooling layer that reduced the volume to a 32-dimensional feature vector. This acted as a compact representation for each time step. The dimensionality of 32 features was chosen based on a standard convolutional design heuristic, in which channel depth was increased as the spatial resolution was reduced. This scheme was followed throughout the SFE, except for in the second block, where the number of channels was intentionally kept low to limit model complexity, given that temporal modeling was deferred to the TFE.

Effectively, each time frame was processed independently by the SFE, exposing the same convolutional filters to each time frame of the dynamic image. In the second part of the model, all the extracted vectors were stacked along the time dimension, over which the temporal feature extractor (TFE) captured temporal correlations of subsequent time frames. The TFE used 1D convolutions, motivated by recent research that has demonstrated superior performance and efficiency compared to alternative methods based on recurrent neural networks [31]. The TFE produced the final model output, that represented the DLIF prediction of the AIF given the input PET image.

### Training procedure

The ADAM optimizer [32, 33] with standard settings was used to minimize the weighted mean squared error (wMSE) between the predicted DLIF and the ground truth. The error on each time step was weighted to account for the imbalance between three parts of the curve: the first 25 (peak), middle 9 (intermediate), and last 8 (tail) frames were associated with weights 0.4, 0.7 and 1 respectively. The weights were empirically determined to approximately balance the uneven temporal resolution. The high granularity of early samples were required to accurately determine the dynamics during the distribution phase of tracer that corresponded to the peak of the input function. Therefore, the curves were weighted roughly inversely proportional to the number of sampling points. Training was performed with a learning rate of 1 $\times 10^{-4}$ for 1000 epochs, which took approximately 3.5 hours on a single GPU. Inference time was measured for a single dynamic PET volume (batch size = 1) on a MacBook Pro (M3, 16 GB RAM) using the PyTorch MPS backend, yielding an average processing time of $0.50\pm 0.01$ s per image. 10-fold cross validation allowed the evaluation of the model performance over the whole training dataset. For each fold, 10 runs were repeated for statistical rigor. Data augmentation with additive random Poisson noise injection were used during training, to expose the model to different signal-to-noise ratios and accustom it to lower image qualities. The degraded image was generated by sampling a scalar $p\sim \textrm{Unif}(0,1)$, then, for a dynamic PET image with voxel intensities *I*, the corresponding image with noise, $I_{\textrm{poisson}}$ was defined as:

1 $$\begin{aligned} \begin{aligned} \Lambda&= I \cdot p\\ I_\textrm{poisson}&\equiv I + \textrm{Pois}(\Lambda ) - \Lambda \end{aligned} \end{aligned}$$

**Fig. 2:**
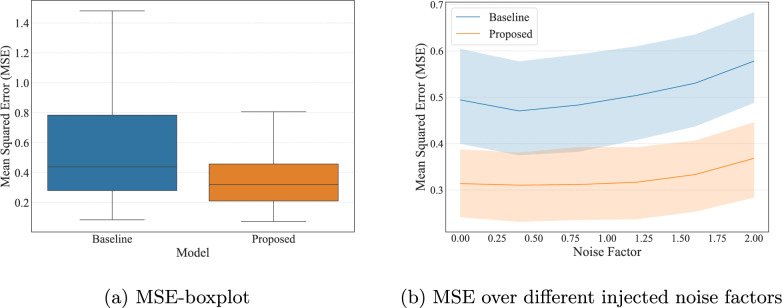
Quantitative comparison between the baseline and the proposed model over each sample, (**a**) boxplot of the MSE from the two models; (**b**) Lineplot with mean and 95% confidence interval for different Poisson noise factors

### Evaluation metrics

#### Quantitative evaluation metrics

Comparisons were done against the former state-of-the-art DLIF model proposed by Kuttner et al. [25], which will be referred to as *the baseline* in the following sections. To ensure fair comparisons, the baseline was trained on the same data as the FC-DLIF, also using 10-fold cross validation.

The predicted DLIF curves were compared time frame by time frame with the respective measured AIF using a paired t-test ($\alpha = 0.05$), and orthogonal regression to account for measurement errors in both predicted and measured variables. An unweighted mean squared error (MSE) was used to directly compare the curves. Normality was assessed using quantile-quantile plots, reporting the Pearson correlation to describe the spread of the predicted curves.

##### Evaluation on downstream tasks

In order to assess the efficacy of the proposed method for downstream tasks, such as kinetic modeling, the graphical Patlak plot [34, 35] was used to produce net influx rates $\textrm{K}_\textrm{i}$ given the reference and predicted input functions for each voxel over time. Prior to kinetic modeling, both the AIF and the DLIF curves were converted to plasma input functions following [36]. The DLIF-based influx estimates were further compared to those obtained from the measured AIF using the same evaluation methods as for the predicted curves.

##### Robustness evaluation of unseen data

Robustness on out-of-distribution (OOD) data containing other tracers ($\mathrm {[^{18}F]FDOPA}$ and $\mathrm {[^{68}Ga]PSMA}$) were performed to quantify the transferability of the FC-DLIF to tracers with other distribution and binding characteristics. These samples were exempt from the training dataset as the model is trained exclusively on PET images of $\mathrm {[^{18}F]FDG}$ infusion.

#### Qualitative evaluation metrics

The best, median and worst predictions of the FC-DLIF model was compared with the true, measured AIF, according to their unweighted MSE scores.

##### Evaluation of versatility

The FC-DLIF networks ability to operate on data with varying temporal dimensions were explored using two tests. First, a time-shifted sample was simulated by prepending the initial time frame to the dynamic PET image, before the radiotracer uptake phase had begun, to reveal any learned time-specific dependencies. For instance, the network might have insisted that time frame $I_t$ always contains the peak of the input function during inference.

Second, the dynamic image was truncated by removing the first 4 frames (first 40 s), and the last 6 time frames (last 30 min) of the signal. This simulated a change in the imaging protocol, where radiotracer infusion and imaging were simultaneously started, partially masking the input function onset, and ending the scan earlier. This experiment was designed to assess the model’s ability to operate with limited scan durations while retaining task performance.

##### Pattern exploration of spatial features

To investigate the latent representations extracted from the SFE, the t-SNE [37] algorithm was used. The algorithm was applied to one model from a single run of a specific fold (fold 2, run 9) to visualize common traits of the condensed spatial representation. The same analysis on the OOD data with other tracers was also done to explore the networks internal representation and understanding of tracers with different properties.

## Results

**Fig. 3:**
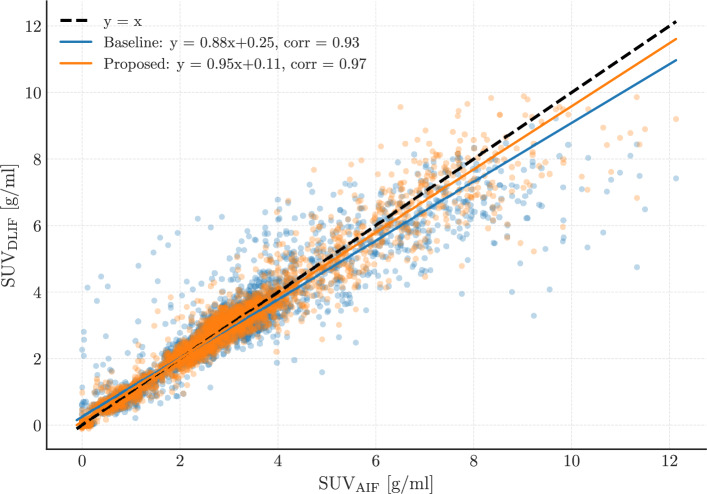
Scatterplot summarizing the results over the whole dataset for the arterial input function estimation for each model. The points show the pointwise comparison between true and predicted values for each mouse, represented by 42 time steps for both the baseline and proposed models. The axes labels indicate the SUV for either the predicted ($\textrm{SUV}_\textrm{DLIF}$), or measured input function ($\textrm{SUV}_\textrm{AIF}$). Ideally for a perfect fit, all points would lie on the black dashed line $y=x$. The coefficient of determination $r^2\le 1$ quantifies how well the predictor fits the data. Similarly, Pearson’s correlation coefficient $r\le 1$ measures the linear correlation between the AIF and the DLIF

First, this section summarizes the results of the proposed FC-DLIF model during cross-validation on our dataset when compared against the baseline, both in terms of errors on AIF estimation and, consequently, after tracer kinetic modeling. Then, some examples of AIF estimation show the performance and versatility of the proposed model with respect to the input data. Finally, the features extracted by the model’s SFE is visualized and inspected using the t-SNE algorithm [37].

### Quantitative results

Figure 2 summarizes the distribution of mean squared error (MSE) (Fig. 2a) and injected noise (Fig. 2b) across all samples. The FC-DLIF consistently achieves lower MSE values than the baseline, with a noticeably narrower spread and a more favorable distribution across percentiles. In terms of noise, the FC-DLIF also demonstrates reduced variability, being less sensitive to noise in the input data.

In particular, the proposed model has close to 75% of MSE values lower than the median of the baseline model, and generally produce predictions more aligned with the AIF, as seen in the $5^{th}$ percentile (best 95% of predictions), which is slightly lower than the baseline (Fig. 2a). These results are further consolidated when comparing the same metrics over temporal regions, as shown in Supplementary Fig. S1, where the proposed method exhibits less bias in most segments.

In Fig. 3, the predicted AIF values from each model are compared against ground truth measurements on a point-wise basis. The proposed method achieves higher $R^2$ and Pearson correlation values, with predictions more closely aligned to the ideal $y = x$ line, indicating a tighter spread of predictions around the regression line. While the baseline exhibits systematic deviations, particularly overestimating intermediate and tail values and underestimating peak amplitudes, the FC-DLIF maintains a tighter spread around the regression line.

Predictions from the baseline model are generally overestimating small values (less than 4 g/mL), and underestimating larger values, associated with the peak of the input function curve. The same characteristics are not seen for the FC-DLIF method, but instead the spread is generally larger above values of 4 g/mL, which is also shown in Supplementary Fig. S1, for the peak labeled segments.

#### Results on downstream tasks

**Fig. 4:**
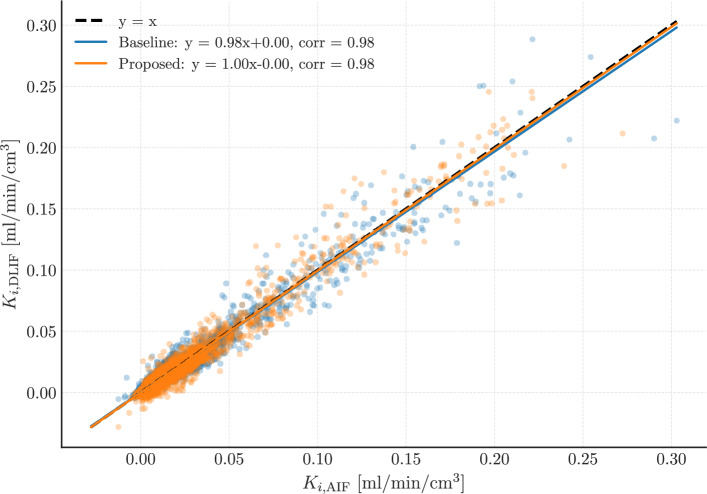
Scatterplot summarizing the results over the whole dataset for voxel-wise tracer kinetic modeling. Each point represents a voxel within a mouse, randomly sampled over 50000 voxels. Also for this figure, the fitted lines should ideally be overlapping the dashed $y=x$ line

Downstream implications of AIF estimation accuracy are reflected in kinetic modeling results shown in Fig. 4, where voxel-wise $K_i$ values derived from each model are compared to the reference values obtained using the measured AIF and the Patlak model [34, 38]. The FC-DLIF again yields higher agreement with the reference, confirming that improvements in input function estimation translate to better physiological parameter estimation.

Analysis using the Patlak plot discards AIF signal from the distribution phase, and assumes a steady-state uptake to be valid for estimating macro-parameters such as $K_i$. The results confirm that even if the peak predictions are imperfect, and exhibit larger errors, the accurate modeling of the linear phase still enables reliable kinetic quantification when using the Patlak plot.

Results from full region-wise kinetic modeling with an irreversible two-tissue compartment model can be found in the supplementary material (myocardium, Fig. S5, and brain, Fig. S6).

#### Robustness to unseen data

Results on the unseen tracer data ($\mathrm {[^{18}F]FDOPA}$ and $\mathrm {[^{68}Ga]PSMA}$) showed decreased performance for both models, as expected due to different tracer-specific uptake characteristics from $\mathrm {[^{18}F]FDG}$ tracers. Additional scatterplots and analyses are provided in the supplementary material (Supplementary Figs. S2–S4).

### Qualitative results

This section evaluates the predictions of the proposed FC-DLIF model. This is followed by an experiment that shifts the PET images in time, delaying tracer injection start. Lastly, the PET images are truncated, simulating simultaneous tracer injection and scan start.

**Fig. 5:**
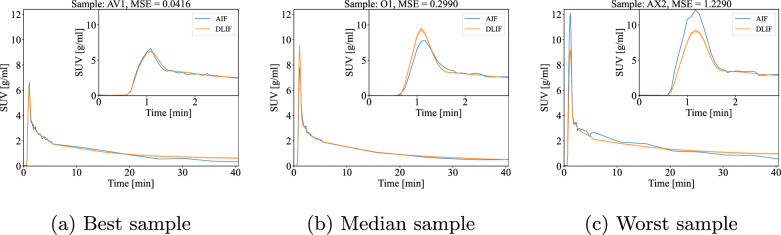
Examples of the input function predicted with the FC-DLIF compared with the ground truth AIF. The insets zoom on the first 3 minutes of the curves, to emphasize the tracer uptake peak. (**a**) Best sample; (**b**) median sample; (**c**) worst sample

Fig. 5 shows three examples of input functions predicted by the proposed FC-DLIF model. The mean curve and standard deviation of the model’s predictions are included for the best (Fig. 5a), median (Fig. 5b), and worst (Fig. 5c) sample according to the MSE.

#### Versatility of the FC-DLIF

**Fig. 6:**
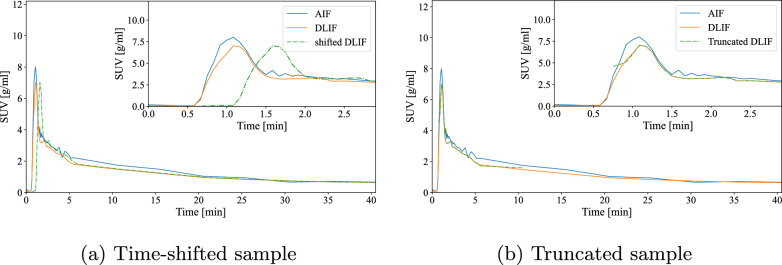
Special cases of input function predicted with the FC-DLIF. The insets zoom on the first 3 minutes of the curves: (**a**) Shifted sample. An additional empty 30 second frame was added at the beginning of the input time series; (**b**) Truncated sample. The first 40 seconds and the last 30 minutes of the input time series were removed, corresponding to the first 4 and last 6 time steps

Fig. 6 displays the versatility of the proposed model owing to its fully convolutional design. In particular, the example in Fig. 6a is the output of the model when the input data is time-shifted by prepending the first frame of the dynamic PET image to itself. The example on the right is the result obtained by truncating the input both at the beginning and at the end of the time series, removing several time steps. The design of the model permits both delays and truncation of the input PET scan without affecting the performance of the predicted AIF.

#### Pattern exploration of spatial features

**Fig. 7:**
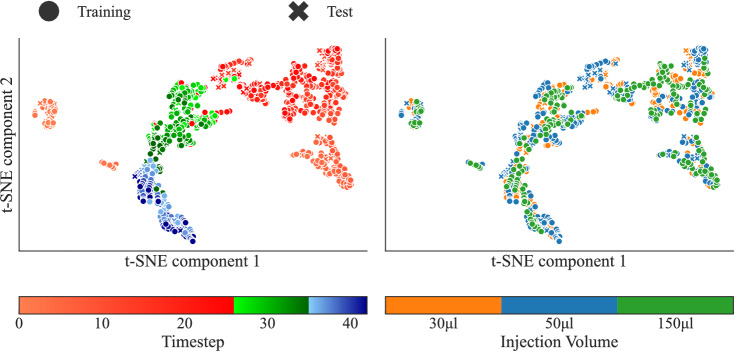
t-SNE visualization of the SFE-extracted vectors for different injection volumes. On the left-hand side, the colormap distinguishes peak (red), intermediate (green), and tail (blue) time steps. To the right, the colormap indicates the various mice groups

**Fig. 8:**
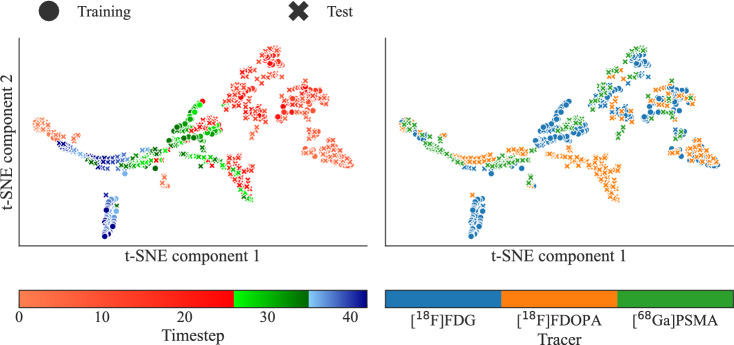
t-SNE visualization of the SFE-extracted vectors for different injected tracers. To the left, the colormap distinguishes peak (red), intermediate (green), and tail (blue) time steps. To the right, the colormap indicates the various mice groups

Finally, the t-SNE is used to visualize the similarities between the high-dimensional vectors extracted by the SFE for each time step of each mouse, on both the training and the test datasets. Fig. 7 and 8 illustrate the results of the t-SNE dimensionality reduction for the groups of injected volume and injected tracers respectively. To the left, the colormap indicates the peak, intermediate, and tail time steps in shades of red, green, and blue respectively. On the right-hand side, the colormap indicate certain attributes: the amount of $\mathrm {[^{18}F]FDG}$ injected (Fig. 7), and what tracer is used (Fig. 8).

Fig. 7 shows a clear temporal progression in the feature space. Early frames with low uptake, likely corresponding to the initial bolus arrival, clusters tightly on the left, followed by a gradual arc through peak (vascular uptake) and post-distribution (tissue uptake) frames. The pattern reflects the biological progression of tracer dynamics and suggests that the SFE captures consistent spatial characteristics across time. This suggests that the SFE successfully encodes temporally meaningful spatial information. Notably, the feature clusters are consistent across both training and test sets, with no clear separation, indicating successful generalization while not overfitting. Furthermore, the feature space shows no meaningful correlation with injected volume, suggesting that the SFE learns to abstract away trivial scaling differences in tracer volume, which is an encouraging sign of biological relevance and robustness.

Likewise, Fig. 8 shows the t-SNE projection of different tracer groups that are not used during training, consisting of $\mathrm {[^{18}F]FDG}$, $\mathrm {[^{68}Ga]PSMA}$, and $\mathrm {[^{18}F]FDOPA}$ injected mice. While the early and peak phases of all tracers appear to follow a similar initial trajectory, clear deviations form in the later frames. In particular, the steady-state representations of the $\mathrm {[^{68}Ga]PSMA}$ and $\mathrm {[^{18}F]FDOPA}$ groups form separate clusters, distinct from the $\mathrm {[^{18}F]FDG}$ tail-phase points. This indicates that the model’s spatial encoder captures consistent tracer-specific signatures, even on samples not seen during training. Additionally, the $\mathrm {[^{18}F]FDOPA}$ group also diverges around the late peak phase, forming a distinct cluster that may explain the reduction in accuracy of the model for this group. This result reflects fundamental differences in tracer kinetics and binding properties [39], and align with the systematic under- and overestimation patterns seen in the early and late phases of predicted AIFs for the OOD tracers. The observed mismatch suggests the model’s learned temporal filters in the TFE are not readily transferable across radiotracers without retraining.

## Discussion

This study introduces the FC-DLIF, a robust and flexible deep learning model for the non-invasive estimation of AIF directly from dynamic PET imaging data. Compared to existing methods, the FC-DLIF demonstrates superior accuracy, reduced bias, and improved robustness to noise and input variability, while maintaining flexibility with respect to temporal shifts and varying scan lengths. The fully convolutional design enables the model to generalize to different imaging protocols without the need for rigid preprocessing or fixed input dimensions. However, robustness evaluations on OOD tracer data with different physiological characteristics confirms that specialized training is required.

Structurally separating the objective into two separate components, the SFE and TFE, allows each part to specialize in extracting relevant representations. Practically, applying the same SFE weights to each input time frame, ties the weights of the SFE over time. This encourages the SFE to represent an early and a late time point differently enough for the TFE to detect. Furthermore, it means that the TFE cannot depend on characteristic cues seen in early or late parts of the PET image. For instance, the smoother appearance in later time frames after the noisy initial distribution phase of tracer, when more detections are collected in each frame. Instead, the model relies on the extracted features of the SFE, that abstracts away minute details (Fig. 7 and 8). This is confirmed by shifting-in-time and truncation of the input image (Fig. 6), which for a fixed input-length network might encode absolute temporal positions, and accordingly, react to missing or altered time frames. The FC-DLIF is agnostic to such alterations of the temporal dimension, learns more generalized temporal patterns of tracer dynamics, improving alignment with the true AIF.

The choice of placing the SFE before the TFE is intentional. It could be argued that similar traits would be possible with the opposite order, placing the TFE before the SFE. An issue with this approach is that the 1D-temporal convolutions of the TFE would then need to extract a meaningful representation directly from the sparse PET images, retaining the 4D representation ($\text {time} \times \text {depth} \times \text {height} \times \text {width}$) through the whole TFE, followed by an SFE concentrating the spatial dimensions into one scalar per frame. This approach requires a lot more memory than first reducing the spatial dimensions into a small vector. Another approach could join the SFE and TFE by alternating spatial and temporal specialized layers. While the temporal-focused layers also needs to apply 1D convolutions to much larger matrices in the initial layers, when the spatial dimensions are large, the separation of tasks are lost. A key benefit of our model relates to how each subnetwork is able to specialize; introducing a form of structural regularization, while staying interpretable (as we have shown with Figs. 7 and 8).

The improvements in AIF prediction directly translate into better physiological parameter estimation, as evidenced by the voxel-wise $K_i$ comparisons (Fig. 4), and region-wise comparisons (Supplementary Fig. S5 and Supplementary Fig. S6). Accurate AIF estimation is critical for computing reliable kinetic parameters using models such as Patlak [34, 38] and compartment models [40]. The enhanced performance of the FC-DLIF further supports its potential to replace invasive blood sampling without compromising modeling accuracy. This effort opens the possibility for instant, and automatic AIF estimation in clinical routine, further lowering procedural requirements for clinical use. For instance, static PET is commonly used in clinical PET on humans, partly due to increased labor and costs associated with AIF estimation, and increased complexity of data analysis. However, our focus is predominantly on preclinical settings, where longitudinal studies in mice is limited by terminal surgical necessary for arterial cannulation. The FC-DLIF allows bypassing invasive measures altogether.

A final key advantage of the FC-DLIF is its compact size. The model end-to-end processes 4D PET volumes yet comprises only 90 124 trainable parameters (approximately 352 KB in FP32 precision), comparable to the baseline of 92 104 parameters (approximately 360 KB in FP32 precision). For reference, the FC-DLIF is over 100 times smaller than a single PET scan (approximately 37 MB in FP32 precision). The compact nature of the algorithm makes it suitable for local use, being approachable on consumer-grade equipment.

### Limitations

When applied to a dataset using tracers not seen during training the model exhibits reduced performance. The observed divergence between the tracers reflects fundamental biological differences (Fig. 8). For instance, $\mathrm {[^{18}F]FDG}$ uptake is driven by perfusion and metabolism, while $\mathrm {[^{18}F]FDOPA}$ is transport- and region-specific, and $\mathrm {[^{68}Ga]PSMA}$ is receptor binding dominated [41]. This leads to distinct vascular-tissue coupling and late-phase spatial signatures, making transferability unfeasible without retraining on similar data. Despite this limitation, the meaningful spatial embeddings on the unseen tracers provides an opportunity for targeted adaptation. For instance, freezing the SFE while training the TFE on a small amount of new tracer data could map the distinct clusters back onto the common manifold of $\mathrm {[^{18}F]FDG}$ (Fig. 8). This strategy aligns with principles from transfer learning [42] and domain adaptation [43], and could substantially reduce the data requirements for successful adoption of the FC-DLIF to new tracers. Since the temporal behavior of the tracers is the culprit of the variation, reusing spatial filters while tailoring the temporal encoder, offers a cheap solution to generalize better. Other future work using techniques such as tracer-aware conditioning, or semi-supervised strategies [44] may improve downstream practicality.

The FC-DLIF does not constrain the temporal dimensions of the input data, however, this is not the case for the spatial dimensions. Given a pretrained model, future studies are required to preprocess the spatial dimensions of their data to match that of the data it was trained on. This criterion is however simple to meet through abundantly available transformations and interpolation algorithms for the spatial dimensions.

While the proposed model demonstrates robustness to temporal shifts and truncated sequences, this study did not investigate how motion-induced misalignment or frame-to-frame deformation may affect the FC-DLIF performance. Convolutional neural networks are translationally equivariant by design, and commonly exhibit approximate translational invariance when combined with pooling and hierarchical feature representations [45, 46]. As a result, small inter-frame spatial perturbations are expected to have limited impact on higher-level feature representations. Additionally, the spatial feature extractor processes each time frame independently using shared convolutional filters, which may further mitigate sensitivity to minor inter-frame deformations. A dedicated evaluation of robustness to respiratory and cardiac motion will be investigated in future studies.

While the FC-DLIF relies on frame-wise reconstructed PET images, it accepts any frame-length as input, making it versatile to the framing chosen during reconstruction of the data. However, an appropriate investigation of reconstruction parameters, framing scheme, PSF modeling, scatter and dead-time corrections, and partial-volume effects, was out of scope for the current study. This could be investigated in future research.

Finally, future work should focus on validating the methodology’s generalization to other imaging subjects than mice, to subjects inhibiting pathologies, and to different imaging systems and modalities.

## Conclusion

This study introduces a non-invasive, deep learning-based input function predictor, that provides a flexible method that is invariant to time-shifting and truncation of the sequence length. Altogether, the FC-DLIF represents a promising tool for small-animal $\mathrm {[^{18}F]FDG}$ PET studies, removing the need for invasive blood sampling and enabling more efficient and scalable experimental designs.

## Supplementary Information

Supplementary Material 1

## Data Availability

The datasets used and analyzed for the study are available from the corresponding author upon reasonable request.

## Abbreviations

PET: Positron emission tomography
FDG: Fluorodeoxyglucose
FC-DLIF: Fully convolutional deep learning-based input function
AIF: Arterial input function
FDOPA: Fluorodopa
PSMA: Prostate-specific membrane antigen
PBIF: Population-based input function
IDIF: Image-derived input function
TAC: Time-activity curve
SIME: Simultaneous estimation
ROI: Region of interest
LSTM: Long short-term memory
SFE: Spatial feature extractor
TFE: Temporal feature extractor
MSE: Mean squared error
wMSE: Weighted mean squared error
DLIF: Deep learning-based input function
t-SNE: t-distributed stochastic neighbor embedding
CT: Computed tomography
OOD: Out-of-distribution
