# Supplementary material for: A robust and versatile deep learning model for prediction of the arterial input function in dynamic small animal [18F] FDG PET imaging
Source: EJNMMI Res. 2026 Mar 9;16:65. doi: 10.1186/s13550-026-01398-9 (PMC13087074; doi:10.1186/s13550-026-01398-9)
Supplement: Supplementary file 1 — Supplementary Material 1 [file 13550_2026_1398_MOESM1_ESM.pdf]

Supplementary Information for "A robust and versatile deep learning model for prediction of the arterial input function in dynamic small animal [^{18}F]FDG PET imaging"

S1 Data acquisition and processing

S1.1 Animal experiments

The imaging data of mice used to train and evaluate the FC-DLIF model were acquired at UiT The Arctic University of Norway (UiT). All animal experiments were approved by the Norwegian Food Safety Authority; FOTS id 29689. In total, 80 healthy female mice from three different strains were included in this study: BALB/cJRj (N = 55), C57BL/6JRj (N = 8) and Balb/cAnNCrl (N = 7), where BALB/cJRj made up the main group of 70 samples. The remaining 10 were set aside for evaluating the effects of the tracers [^{18}F]FDOPA (N = 6) and [^{68}Ga]PSMA (N = 4). The mice were 7 to 8 weeks of age upon arrival to the UiT animal facility, and fed ad libitum a standard rodent diet.

S1.2 Arterial and venous cannulation during image acquisition

At the time of PET/CT imaging, the mouse age was 13.0 ± 0.9 weeks, with a corresponding weight of 22.5 ± 0.5 g. The study used three different radiotracers: [^{18}F]FDG, [^{18}F]FDOPA, and [^{68}Ga]PSMA. The mice receiving [^{18}F]FDG (70 animals) were fasted for 3.4 ± 0.2 hours prior to injection, whereas the remaining animals—given [^{18}F]FDOPA and [^{68}Ga]PSMA—were not fasted. The mice were anesthetized prior to the scan, weighed, and placed on a heated plate (38 °C) while receiving oxygen through a mask. A venous catheter was inserted into the tail vein of each mouse for radiotracer injection. The blood glucose during venous cannulation was measured to $6.4 \pm 0.3\,\mathrm{mmol\,L^{-1}}$. An incision in the neck enabled surgical cannulation of the carotid artery, allowing blood to be routed through a radiation detector at a withdrawal rate of $105.6 \pm 2.8\,\mathrm{\mu L\,min^{-1}}$. This setup facilitated concurrent measurements of whole blood activity during the PET scan with a temporal resolution of 1 s. To enable continuous arterial line measurements, an arterial–venous shunt was established, forming a closed loop between arterial sampling and venous reinjection. Blood flowed sequentially from the arterial sampling line, radiation detector, a peristaltic pump, and a Y-connector—enabling intravenous radiotracer injection—before being reinfused via the venous injector, thereby preventing excessive blood loss.

The PET/CT imaging was performed using a 45.5 min listmode scan on a Triumph^{TM} LabPET-8^{TM} small animal PET/CT scanner (TriFoil Imaging Inc., Chatsworth, CA,

USA) while a sensor monitored the respiration rate. The mice were injected with 16.2 ± 0.7 MBq using an automated injection pump, started 30 s after scanning was initiated. CT imaging was performed after PET scanning to correct for attenuation and scatter. While still under deep anesthesia after scanning, the mice were euthanized using cervical dislocation. Scanner sensitivity was monitored through daily phantom calibrations.

S1.3 Calibration and AIF processing

To allow for delay correction of the manual blood sample measurements in the arteriovenous shunt, the time delay between the radiation detector and the Y-connector was measured during the first pass of arterial blood to 25.1 ± 0.7 s. The continuous line radiation detector was calibrated with three manual blood samples taken in a late stage or post-scan by measuring blood dripped from the arterial catheter over 30 s. This also enabled measurement of the actual arterial withdrawal rate during each mouse scan.

A calibration factor for the continuous arterial line measurements was derived as the ratio of the average signal from the continuous line measurements and the manual blood samples collected during the same 30 s blood sampling at each time point, corrected for delay. Calibration factors outside three scaled median absolute deviations from the median factors were considered outliers and discarded [Hubert and Van der Veeken (2008)]. An overall calibration factor was determined as the average factor from the included blood sample factors. The arterial input function (AIF) for each mouse was obtained by scaling the continuous line measurement data by this average calibration factor.

S1.4 Image reconstruction and processing

The PET images were reconstructed into 42 time frames (1×30 s, 24×5 s, 9×20 s and 8×300 s) using a three-dimensional maximum-likelihood estimator algorithm with 50 iterations. Corrections for detector efficiency, radioactive decay, random coincidences, dead time, attenuation, and scatter were applied. Each time frame had an image matrix size of $128 \times 92 \times 92$ voxels. The voxels were converted from units of counts per second into units of MBq mL^{-1} using the average counts inside a 14 mL homogeneous image region of a daily phantom scan. Subsequently, the voxels were normalized into standardized uptake value (SUV) [g mL^{-1}] [Keyes (1995)].

S2 Additional results

S2.1 Detailed results for individual imaging phases

Figure S1 visualizes the distribution of error between the predicted input function from the baseline model [Kuttner et al. (2024)] and FC-DLIF compared with the AIF, as a function of time since the start of tracer injection. A predominantly negative distribution in the plot would indicate the model underestimating the AIF.

S2.2 Results on unseen tracers dataset

Figure S2 compares the baseline [Kuttner et al. (2024)] and the proposed FC-DLIF models' SUV predictions with the measured AIF. The lines are computed as the orthogonal

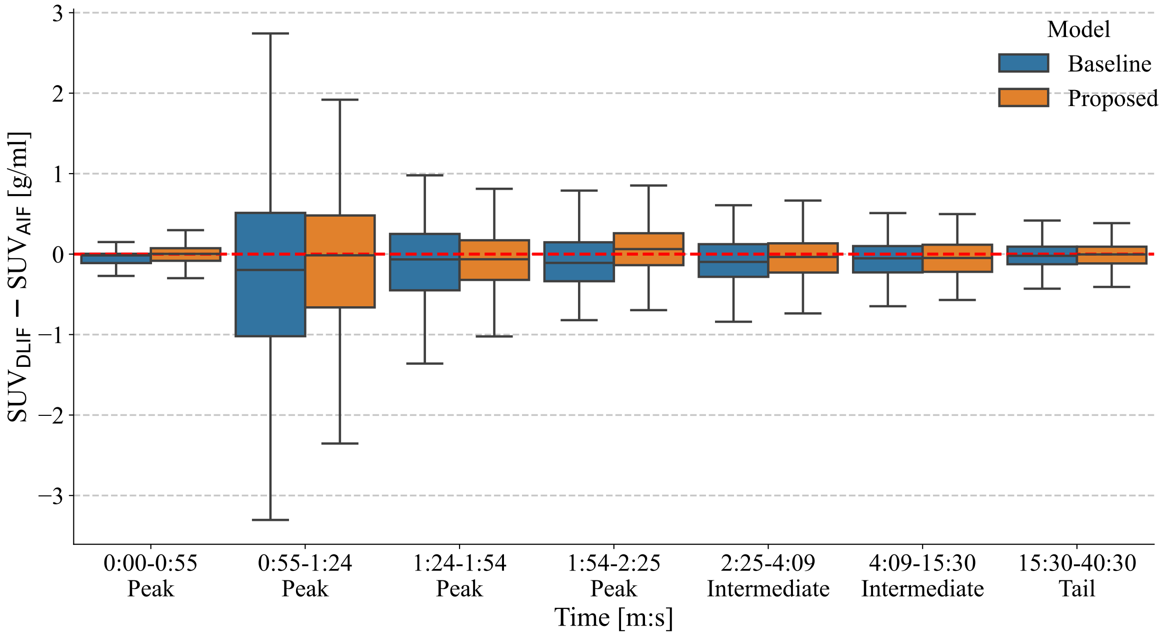

Figure S1: Prediction error distribution during different phases of the imaging for the baseline DLIF [Kuttner et al. (2024)] and the proposed FC-DLIF.

regression line fitted to the point distributions of each respective color. A perfect predictor would have SUV points distributed across the black, dashed, $y = x$ line.

Figure S3 uses the predicted input function from either model in a Patlak graphical analysis [Patlak et al. (1983), Patlak and Blasberg (1985)] to derive the kinetic parameters K_i.

Figure S4 shows examples of input functions predicted by the proposed FC-DLIF model. The mean curve and the standard deviation over the 10 runs are depicted for the best (left), median (middle), and worst (right) sample according to the mean squared error (MSE).

S2.3 Detailed kinetic modeling results

Further kinetic modeling results using the predicted input functions from both models are summarized in Figures S5 and S6. The kinetic parameters were estimated using an irreversible two-tissue compartment model [Sokoloff et al. (1977)] in two different regions: myocardium and brain. The estimated parameters were compared with those obtained using the measured AIF and DLIF. The quantile–quantile plots show the distribution of the kinetic parameters estimated using orthogonal regression.

Compared to the Patlak analysis presented in the main manuscript, the kinetic parameters derived from the two-tissue compartment model are less stable because the full measurement period is used in the fitting, and not only the linear phase. Outliers are therefore removed if they lie more than three standard deviations away from the mean of each parameter distribution. Once an outlier is detected, it is removed from all parameters in the respective region for consistency. This results in 64 and 66 samples remaining for the myocardium and brain regions, respectively.

The observed myocardium kinetic parameters (Figures S5 and S5e) follow the ref-

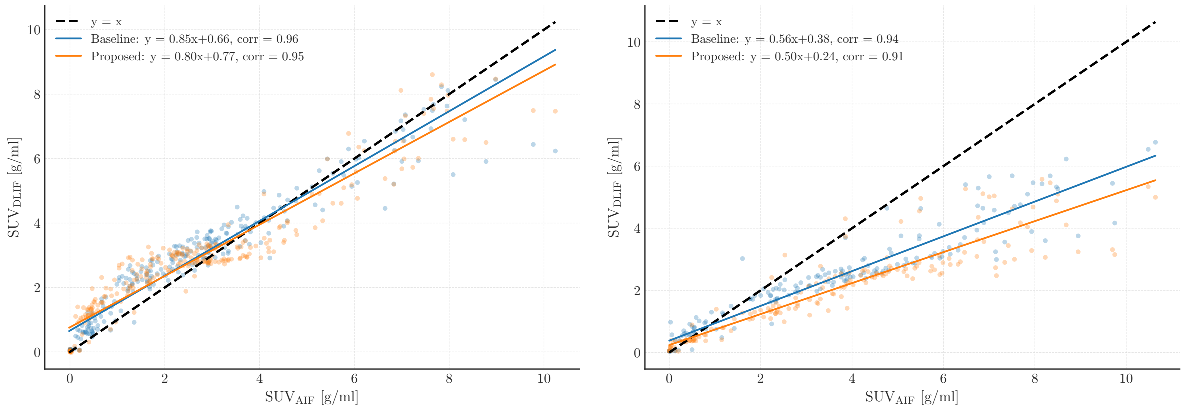

(a) FDOPA (b) PSMA

Figure S2: Scatterplots summarizing the results over the unseen tracers dataset for the arterial input function estimation. The color represents SUV predictions for each model.

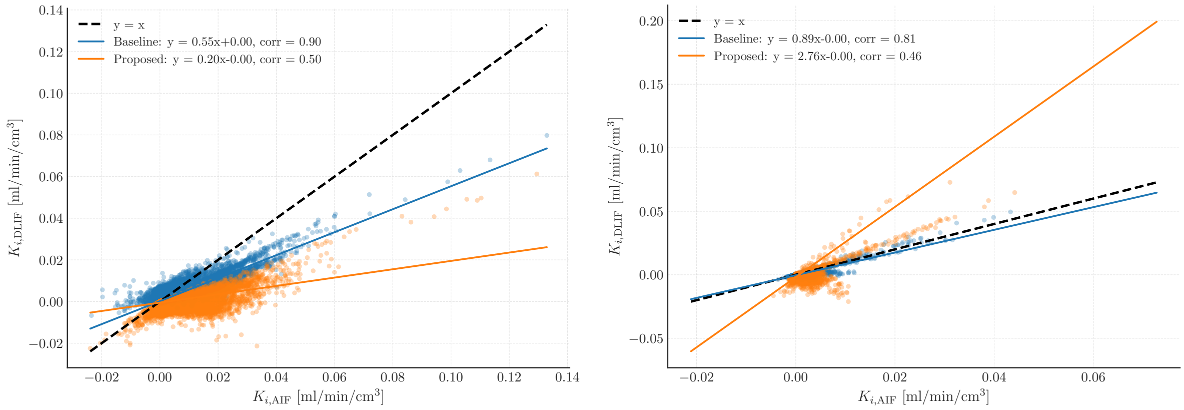

(a) FDOPA (b) PSMA

Figure S3: Scatterplots summarizing the results over the unseen tracers dataset for voxel-wise tracer kinetic modeling. Colors indicate which model provided each coefficient for a random subset of 50 000 voxels.

erence distribution well, with larger spread in k_2, k_3, and V_b, as well as outliers not removed by the three standard deviation rule in k_2 and the blood volume fraction. Influx rates (Figure S5e) were higher than previously reported [Kreissl et al. (2011), Wong et al. (2011)]. This is likely due to shorter fasting times in our study, where longer fasting times are known to reduce influx rates in the myocardium [Kreissl et al. (2011), Wong et al. (2011)].

The parameter distributions in the brain (Figure S6) had fewer outliers, and similar to the myocardium, k_2, k_3, and V_b had larger spread than the uptake and influx rate. The ranges of the brain kinetic parameters are in line with previous literature [Alf et al. (2013)].

References

[Hubert and Van der Veeken (2008)] Hubert M, Van der Veeken S. Outlier detection for skewed data. J Chemom. 2008 3;22(3-4):235–246. https://doi.org/10.1002/cem.

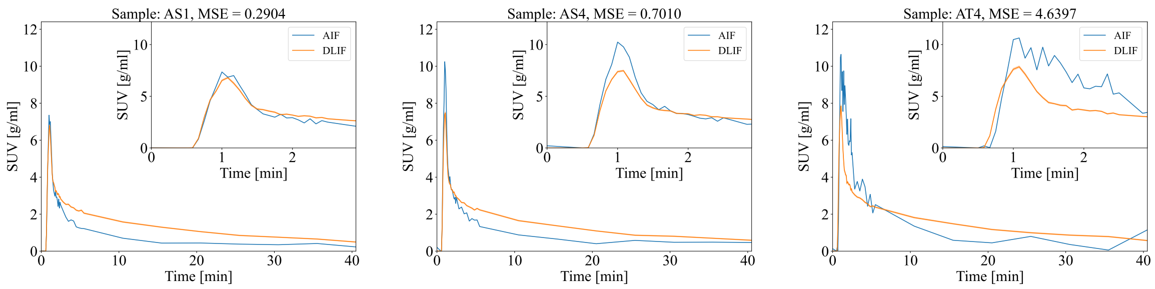

(a) Best sample (b) Median sample (c) Worst sample

Figure S4: Examples of input function predictions with FC-DLIF compared against the ground truth on unseen tracers. Insets zoom on the first 3 minutes of the curves, when the input function peak occurs. (a) Best sample ([^{18}F]FDOPA); (b) median sample ([^{18}F]FDOPA); (c) worst sample ([^{68}Ga]PSMA).

1123.

[Keyes (1995)] Keyes JW. SUV: standard uptake or silly useless value? J Nucl Med. 1995;36(10):1836–1839.

[Kuttner et al. (2024)] Kuttner S, Luppino LT, Convert L, Sarrhini O, Lecomte R, Kampffmeyer MC, et al. Deep-learning-derived input function in dynamic [18F]FDG PET imaging of mice. Frontiers in Nuclear Medicine. 2024;4. https://doi.org/10.3389/fnume.2024.1372379.

[Patlak et al. (1983)] Patlak CS, Blasberg RG, Fenstermacher JD. Graphical Evaluation of Blood-to-Brain Transfer Constants from Multiple-Time Uptake Data. Journal of Cerebral Blood Flow and Metabolism. 1983;3:1–7. https://doi.org/10.1038/jcbfm.1983.1.

[Patlak and Blasberg (1985)] Patlak CS, Blasberg RG. Graphical evaluation of blood-to-brain transfer constants from multiple-time uptake data. Generalizations. Journal of cerebral blood flow and metabolism : official journal of the International Society of Cerebral Blood Flow and Metabolism. 1985 12;5:584–90. https://doi.org/10.1038/jcbfm.1985.87.

[Sokoloff et al. (1977)] Sokoloff L, Reivich M, Kennedy C, Rosiers MHD, Patlak CS, Pettigrew KD, et al. The [14C]Deoxyglucose Method for the Measurement of Local Cerebral Glucose Utilization: Theory, Procedure, and Normal Values in the Conscious and Anesthetized Albino Rat. Journal of Neurochemistry. 1977;28(5):897–916. https://doi.org/10.1111/j.1471-4159.1977.tb10649.x.

[Kreissl et al. (2011)] Kreissl MC, Stout DB, Wong KP, Wu HM, Caglayan E, Ladno W, et al. Influence of dietary state and insulin on myocardial, skeletal muscle and brain [18F]-fluorodeoxyglucose kinetics in mice. EJNMMI research. 2011;1(1):8. https://doi.org/10.1186/2191-219X-1-8.

[Wong et al. (2011)] Wong KP, Sha W, Zhang X, Huang SC. Effects of administration route, dietary condition, and blood glucose level on kinetics and uptake of 18F-FDG in mice. Journal of Nuclear Medicine. 2011;52(5):800–807. https://doi.org/10.2967/jnumed.110.085092.

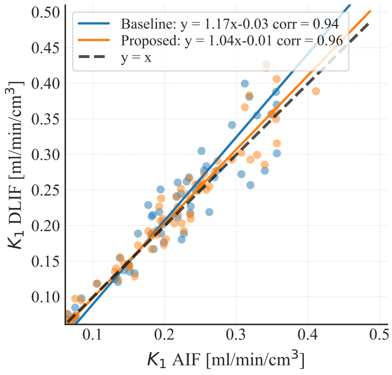

(a) K_1

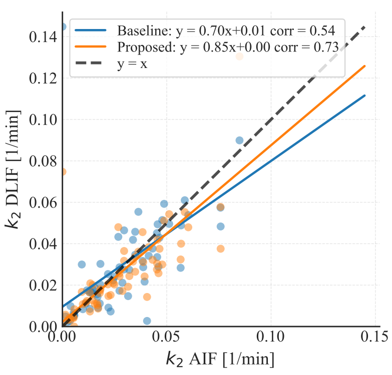

(b) k_2

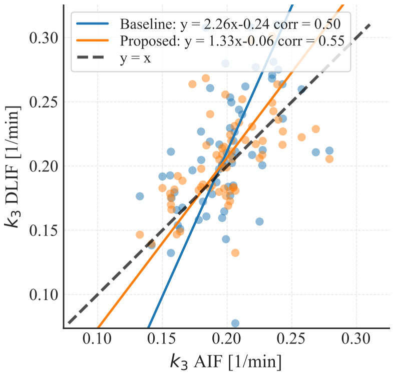

(c) k_3

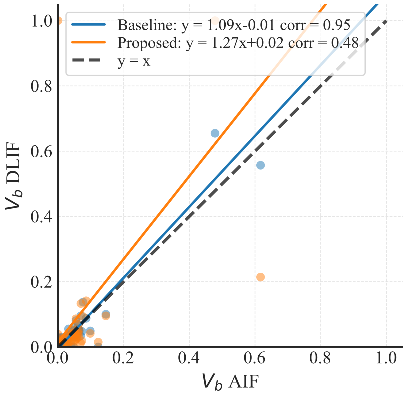

(d) V_b

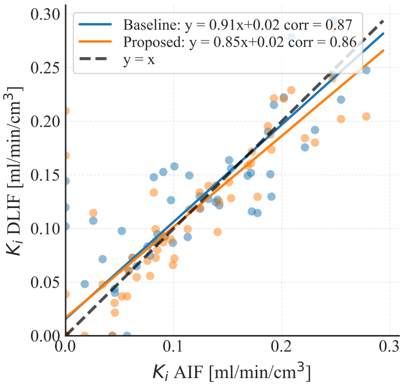

(e) K_i

Figure S5: Scatterplots summarizing kinetic parameters estimated using an irreversible two-tissue compartment model in the myocardium region. Points represent each region in each mouse sample, colored by the model producing the coefficient. From top left to bottom right: K_1, k_2, k_3, V_b, and K_i.

[Alf et al. (2013)] Alf MF, Martić-Kehl MI, Schibli R, Krämer SD. FDG kinetic modeling in small rodent brain PET: optimization of data acquisition and analysis. EJNMMI research. 2013;3(1):61. `https://doi.org/10.1186/2191-219X-3-61`.

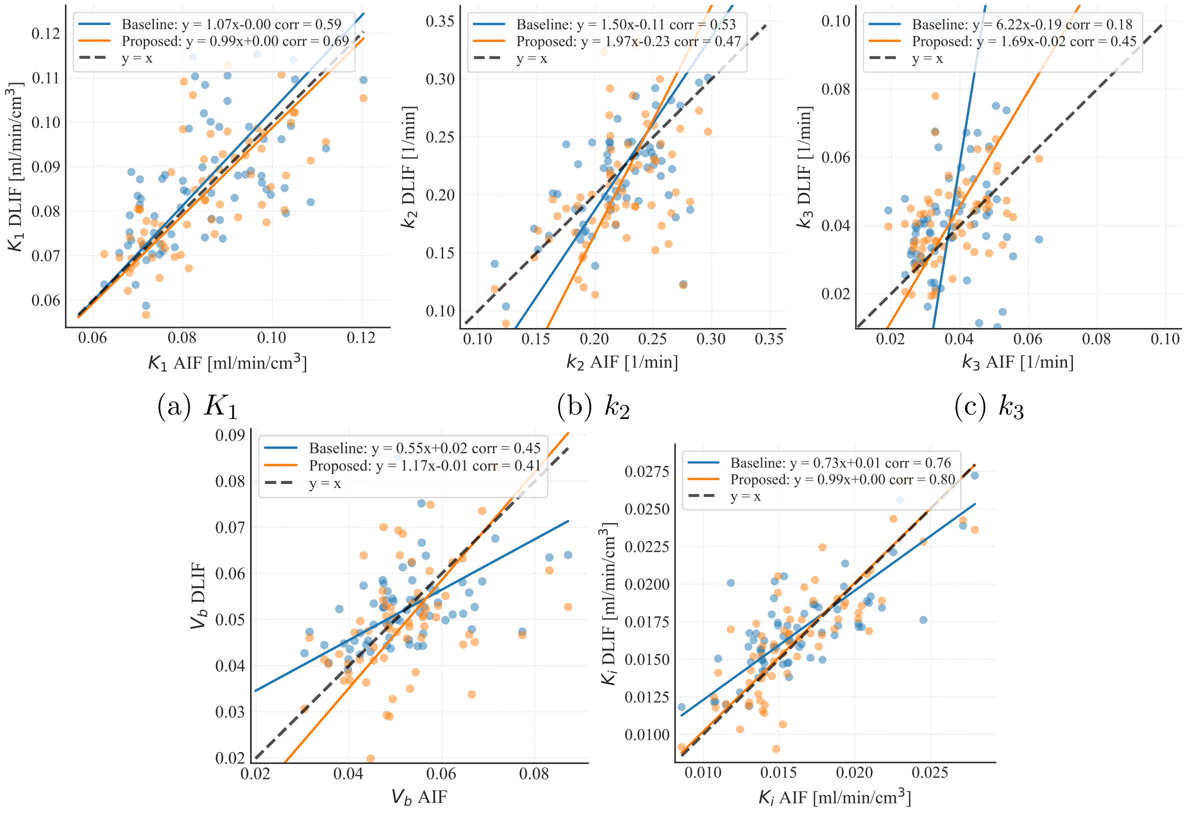

(a) K_1 (b) k_2 (c) k_3

(d) V_b (e) K_i

Figure S6: Scatterplots summarizing kinetic parameters estimated using an irreversible two-tissue compartment model in the brain region. Points represent each region in each mouse sample, colored by the model producing the coefficient. From top left to bottom right: K_1, k_2, k_3, V_b, and K_i.
